# Supplementary material for: The leader proteins of Theiler’s virus and Boone cardiovirus use a combination of short linear motifs (SLiMs) to target RSK kinases to the nuclear pore complex
Source: J Virol. 2025 Sep 10;99(10):e01275-25. doi: 10.1128/jvi.01275-25 (PMC12548433; doi:10.1128/jvi.01275-25)
Supplement: Fig. S1 — L-induced NCTD kinetics. [file jvi.01275-25-s0001.pdf]

Appendix Fig A1. **L proteins mediate an early nucleocytoplasmic trafficking disorder**

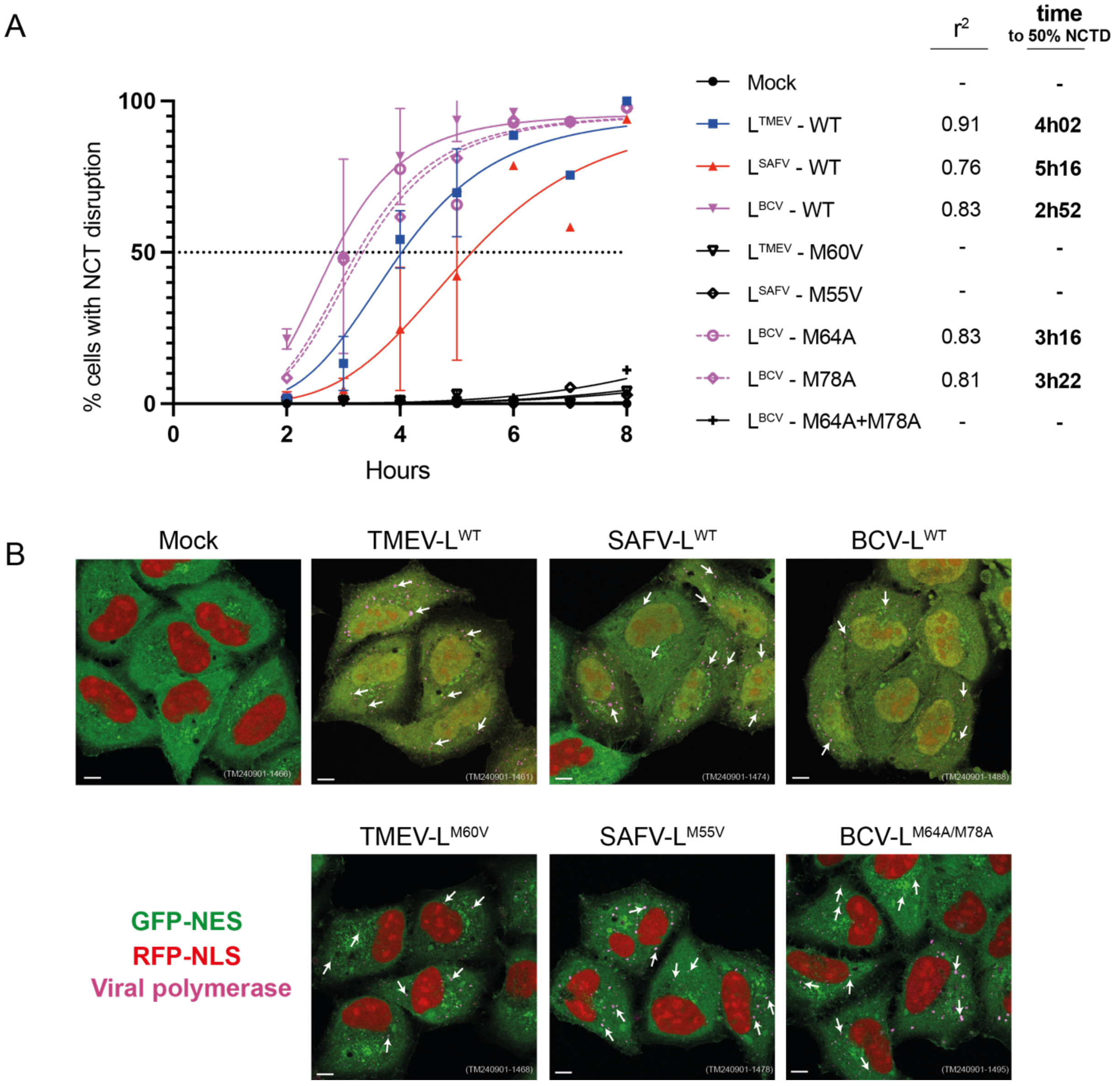

Appendix Fig A1. **L proteins mediate an early nucleocytoplasmic trafficking disorder**

A. Kinetics of NCTD detection in HeLa cells expressing GFP-NES and RFP-NLS infected with 2.5 PFU/cell of recombinant TMEV expressing TMEV, BCV, or SAFV leader proteins and indicated mutants. Data pooled from 3 independent infection experiments were fitted to non-linear curves using Graphpad Prism 10. r^2 values for fitting are indicated as well as the time to reach 50% of NCTD in the cell population. From other experiments, it is considered that >90% of the cells were infected in the cells and the conditions used.

B. Confocal microscopy of HeLa cells expressing GFP-NES and RFP-NLS infected with TMEV derivatives expressing TMEV L, SAFV L or BCV L for 12 h at an MOI of 5 PFU/cell. Cells were fixed and viral polymerase (3D) was immunolabeled as control of infection (purple dots + arrows). Scale bar: 10µm.
